# Short-Term Memory and Long-Term Memory are Still Different

**DOI:** 10.1037/bul0000108

**Published:** 2017-05-22

**Authors:** Dennis Norris

**Affiliations:** 1 MRC Cognition and Brain Sciences Unit, Cambridge, United Kingdom

**Keywords:** long-term memory, memory, STM, working memory

## Abstract

A commonly expressed view is that short-term memory (STM) is nothing more than activated long-term memory. If true, this would overturn a central tenet of cognitive psychology—the idea that there are functionally and neurobiologically distinct short- and long-term stores. Here I present an updated case for a separation between short- and long-term stores, focusing on the computational demands placed on any STM system. STM must support memory for previously unencountered information, the storage of multiple tokens of the same type, and variable binding. None of these can be achieved simply by activating long-term memory. For example, even a simple sequence of digits such as “1, 3, 1” where there are 2 tokens of the digit “1” cannot be stored in the correct order simply by activating the representations of the digits “1” and “3” in LTM. I also review recent neuroimaging data that has been presented as evidence that STM is activated LTM and show that these data are exactly what one would expect to see based on a conventional 2-store view.

For more than a century most psychologists have accepted that there are distinct memory systems responsible for long and short-term storage. Originally based entirely on introspection (e.g., James, 1890), the idea that there are separate long- and short-term memory (LTM and STM, respectively) systems subsequently became a core assumption of modern cognitive psychology. From the 1960s most cognitive models of memory have assumed that there are separate stores (Atkinson & Shiffrin, 1968; Baddeley, 1986; Baddeley & Hitch, 1974; Brown, Preece, & Hulme, 2000; Burgess & Hitch, 1992, 2006; Lewandowsky & Farrell, 2000; Page & Norris, 1998a, 1998b, 2009; Waugh & Norman, 1965). This remains the framework guiding almost all cognitive work on verbal STM.

There are dissenting voices. A number of authors have argued that the behavioral data are more parsimoniously accounted for by assuming that there is only a single memory system responsible for both short- and long-term storage (Brown, Neath, & Chater, 2007; Cowan, 1988, 1999; Crowder, 1993; Crowder & Neath, 1991; Engle, Kane, & Tuholski, 1999; Gruneberg, 1970; Jonides et al., 2008; McElree, 2006; Melton, 1963; Nairne, 2002; Poirier & Saint-Aubin, 1996; Surprenant & Neath, 2009). Some have argued that STM is nothing more than activated LTM (Cowan, 1988, 1999; Oberauer, 2002, 2009). This conception of the relation between STM and LTM has become increasingly popular in neuroimaging research (Acheson, Hamidi, Binder, & Postle, 2011; Cameron, Haarmann, Grafman, & Ruchkin, 2005; D’Esposito & Postle, 2015; Jonides et al., 2008; LaRocque et al., 2015; Postle, 2006; Ranganath & Blumenfeld, 2005; Ruchkin, Grafman, Cameron, & Berndt, 2003). However, there have been no attempts to present an updated case for a multicomponent view of memory in which there is a clear theoretical and conceptual distinction between LTM and short-term stores. Most of the papers cited above have taken a data-driven approach to the debate, in which involves cataloguing similarities between LTM and STM. Here I take a very different approach and consider the computational requirements of a system that could support retention of information over the short term. These impose strong constraints on the architectural relationship between LTM and STM. The overall conclusion will be that, although LTM has an essential role to play, STM cannot be supported simply by activating LTM. Even simple tasks such as remembering a sequence of digits can only be performed by supplementing LTM with some additional mechanism. This additional mechanism is not part of LTM, but has all of the properties typically ascribed to a separate short-term store. Although I will focus primarily on verbal STM, the underlying logic of the arguments applies equally well to STM in other domains. This article begins with a brief review of the core data supporting a distinction between long- and short-term stores. This is followed by an analysis of cognitive theories that have proposed that STM is activated LTM. The concern here will be to establish exactly what the substantive claims of such theories are. I then review the neuroimaging data that have been presented as support for the activation view. In the second half of the paper I focus on computational considerations that argue strongly against the idea that STM might be nothing more than activated LTM. The central problem for activation-based models is that STM has to be able to store arbitrary configurations of novel information. For example, we can remember novel sequences of words or dots in random positions on a screen. These cannot possibly have preexisting representations in LTM that could be activated. The digit sequence 133646 might activate LTM representations of the digits 1, 3, 6 and 4, but some extra mechanism is required to encode the order of the digits and the fact that there are two tokens of the digits 3 and 6. Activating the LTM representations of 1, 3, 6 and 4 is not enough. STM can also store more complex structural representations to encode novel objects and the relation between them. Any viable model of STM must therefore be endowed with additional processes beyond simple activation of LTM. At the very least there must be a mechanism capable of storing multiple tokens, recalling those tokens in the correct order, and creating novel structured representations that cannot yet be in LTM.

I will also ask what it is that we actually store in STM. Does STM contain copies of representations or pointers to representations? If STM contains pointers, do those pointers address LTM, or might those pointers address representations held within the short-term storage system itself? I suggest that, at some level, STM must make use of pointers, but that there are strong computational advantages to having pointers operate within the bounds of a modality-specific STS such as the phonological store component of Baddeley and Hitch’s (1974) Working Memory model. That is, consideration of how a pointer-based system might work provides further support for the idea that there must be a functional distinction between short- and long-term stores.

## Overview

**Figure 1 fig1:**
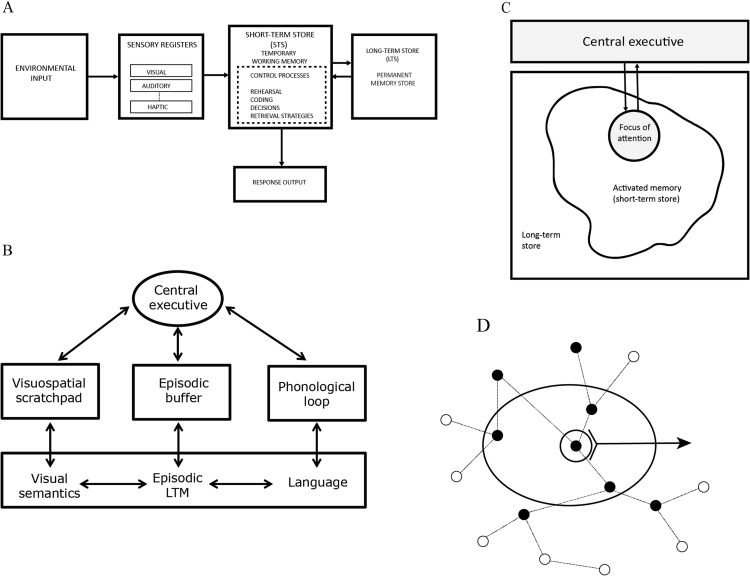
(A) Atkinson and Shiffrin’s (1971) model of STM. (B) Baddeley and Hitch’s working memory model (Baddeley, 2000). (C) Cowan’s (1988) model of memory and attention. (D) Oberauer’s (2002) model of working memory. Nodes and lines correspond to long-term memory representations. Black nodes are activated. Nodes within the large oval are in the direct access region. One node is in the focus of attention.

The idea that STM is merely activated LTM is usually pitted against the classic idea that STM consists of a buffer (or buffers) that holds copies of the items in memory. This view of memory is implicit in Atkinson and Shiffrin’s (1966, 1968, 1971) modal model. Their, 1971 paper begins: “Memory has two components: short-term and long-term” (p. 3). The Atkinson and Shiffrin model is shown in Figure 1A. Figure 1B shows what is probably the most influential multistore model—the Working Memory model of Baddeley and Hitch (Baddeley, 2000; Baddeley & Hitch, 1974). I will present a very brief summary of the findings that have been taken as evidence for a separation between a long-term store (LTS) and a short-term store (STS). The primary data come from studies of STM patients. These patients typically have grossly impaired verbal STM capacity of perhaps 2–4 items, combined with relatively intact LTM (Basso, Spinnler, Vallar, & Zanobio, 1982; Saffran & Marin, 1975; Shallice & Vallar, 1990; Shallice & Warrington, 1970; Vallar & Baddeley, 1984; Vallar, Di Betta, & Silveri, 1997; Vallar & Papagno, 1995; Vallar, Papagno, & Baddeley, 1991; Warrington, Logue, & Pratt, 1971; Warrington & Shallice, 1969). Where it has been examined, these patients generally have few problems with visuospatial STM tasks. The opposite pattern has also been observed. Hanley, Young, and Pearson (1991) reported a patient with impaired visuospatial STM but normal verbal STM. One source of evidence that STM patients have intact LTM is that, in general, these patients have little difficulty in learning new associations between familiar stimuli. However, they do have problems in learning new words (Baddeley, Papagno, & Vallar, 1988; Basso et al., 1982; Bormann, Seyboth, Umarova, & Weiller, 2015; Dittmann & Abel, 2010; Freedman & Martin, 2001; Trojano & Grossi, 1995; Trojano, Stanzione, & Grossi, 1992). The standard interpretation of this finding is that the complete phonological representation of new words must be held in a short-term store in order to be encoded into LTM. Indeed, one of the central functions of STM is assumed to be to hold representations that do not yet exist in LTM (Baddeley, Gathercole, & Papagno, 1998). Importantly, the constituent parts of these representations (perhaps features, phonemes or syllables) must be stored in the correct order. Not surprisingly, these patients also have problems in learning the order of new digit sequences (Bormann et al.). The same logic applies; the sequence must be held in STM so that a representation of the sequence can be presented to LTM.

Conversely, there are patients with medial-temporal lobe damage who have impaired LTM but relatively preserved STM (Baddeley & Warrington, 1970; Cave & Squire, 1992; Drachman & Arbit, 1966; Scoville & Milner, 1957; Wilson & Baddeley, 1988). Ranganath and Blumenfeld (2005) expressed concerns over whether the patient data really do provide clear evidence for distinct stores. They drew attention to the fact that LTM patients with medial temporal lobe damage have difficulties with some STM tasks, and suggest that this “questions whether theories of memory need to propose neurally distinct stores for short- and long-term retention” (p. 374). However, in a later review of the literature, Jeneson and Squire (2012) pointed out that the impairment of LTM patients in STM tasks is only observed with supraspan stimuli that exceed the capacity of STM. Under these circumstances even neurologically normal individuals will necessarily have to rely on a combination of STM and LTM, so it should be no surprise that LTM patients have problems. Furthermore, the finding that LTM patients are sometimes impaired in STM tasks is fully consistent with the two-store view; short-term recall can clearly benefit from a contribution from LTM, even if the two are functionally separate. For example, according to redintegration accounts (Gathercole, Pickering, Hall, & Peaker, 2001; Hulme et al., 1997; Lewandowsky & Farrell, 2000; Saint-Aubin & Poirier, 1999a; Schweickert, 1993; Schweickert, Chen, & Poirier, 1999), information in LTM can be used to reconstruct degraded traces in an STS at recall. Thus, even though LTM may play no role in the maintenance of information in STS, it can potentially improve performance in STM tasks by aiding retrieval.

The most powerful behavioral evidence for a form of distinct short-term storage comes from the early work of Baddeley and colleagues (Baddeley, 1966a, 1966b; Baddeley & Ecob, 1970), which led to the development of the concept of a phonological store. The phonological store is assumed to hold speech-based representations and to have duration of just a few seconds. Information within the store can be refreshed by an articulatory control process (rehearsal) so as to prevent decay, and that same process can be used to recode visual information into a phonological form (Baddeley, Lewis, & Vallar, 1984). Together these for the phonological loop component of the Working Memory model (Figure 1B).

The critical evidence for a phonological store with a limited duration is that while memory confusions at short retention intervals are primarily phonological in nature, confusions at longer retention intervals tend to be semantic. In immediate serial recall, phonological similarity impairs order recall and can even improve item recall (Fallon, Groves, & Tehan, 1999) but semantic similarity has no impact on order recall (Saint-Aubin & Poirier, 1999b; Saint-Aubin & Poirier, 2000). Furthermore, when the items to be remembered are presented visually as opposed to auditorily, the phonological confusions can be eliminated if participants are required to perform articulatory suppression during presentation (Baddeley et al., 1984; Murray, 1968; Peterson & Johnson, 1971). Articulatory suppression is assumed to occupy the articulatory control process and prevent it from being able to recode the visual input into a phonological form.

These findings provided the motivation for separate phonological and visual short-term stores in Baddeley and Hitch’s (1974) Working Memory (WM) model. More recently Baddeley (2000) has expanded the working memory framework to include a component called the episodic buffer (Figure 1B). The episodic buffer is responsible for temporary storage beyond the capacity of the model’s short-term storage systems, and for binding representations across different modalities. One line of evidence supporting the need to add yet another memory system comes from studies of prose recall. For example, the STM patient PV had an auditory word span of one but could remember up to five words when they formed a meaningful sentence (Vallar & Baddeley, 1984).

The strongest evidence for a separate STS does not require carefully controlled laboratory experiments at all. The mere fact that we can repeat nonsense words like ‘blontestaping’ that we have never heard before shows that memory cannot be based simply on activating preexisting representations in LTM. Although there may well be preexisting representations of phonemes, features or syllables, these are not sufficient to construct a mnemonic trace of a previously unencountered nonsense word.

### Short-Term Memory and Working Memory

The terms STM and working memory (WM) are often used interchangeably, and also inconsistently. In this article the primary focus is on whether there are distinct short- and long-term stores. Working memory is usually thought of as a much broader concept, often encompassing processing as well as storage. For example, both the Baddeley and Hitch (1974) and the Cowan (1988) WM models include a central executive component which can operate on the contents of STM (see figure). In the Baddeley and Hitch model, modality-specific short-term stores form part of the overall WM system. The distinguishing feature of WM is that it provides a ‘mental workspace’ (Logie, 2003) that can hold information in a temporary form that can be manipulated and updated. Although there are different views on exactly what constitutes WM (for review see: Aben, Stapert, & Blokland, 2012), the common feature of these views is that rather than being simply a passive store, WM is a system that allows information to be actively manipulated. For example, in the context of visual Luck and Vogel (2013) defined WM “as the active maintenance of visual information to serve the needs of ongoing tasks” (p. 392). Baddeley (1992) described WM as a “system that provides temporary storage and manipulation of the information necessary for such complex cognitive tasks as language comprehension, learning, and reasoning.” (p. 556).

Although this distinction between passive stores and an active mental workspace is widely observed, some authors also use the broader term WM when describing studies that involve only passive storage. This is particularly so in the case of work on visual STM. In contrast, Nee and Jonides (2013) use the term STM to refer to all memory over the short term, whether active or passive. This includes the ‘activated LTM’ component of Oberauer’s (2002, 2009) three-state model. One would more commonly expect this broader conception to be referred to as WM. Ericsson and Kintsch (1995) add further complexity by proposing that there is a form of long-term WM. Although these terminological inconsistencies can lead to confusion, our concern here is with the nature of the storage mechanisms themselves rather than the higher level cognitive processes that might operate of the contents of such stores. Nevertheless, it is worth pointing out that WM tasks like reasoning or language comprehension will necessarily recruit wide-ranging neural and cognitive processes that will simultaneously engage both short- and long-term storage systems.

### Interactions Between STM and LTM

Although multistore models have distinct short- and long-term stores, this does not imply that there are distinct short- and long-term tasks that tap exclusively into short- or only long-term memory. Atkinson and Shiffrin (1968) wrote that “According to our general theory, both STS and LTS are active in both STS and LTS experiments” (p. 101). There is no particular point in time that would mark a transition between tasks that only involve an STS and those that involve only an LTS. Even under the view that there are separate short and long-term storage systems, the two systems should operate in concert. Interactions between LTM and STM have already been mentioned in the context of patients with deficits in STM or LTM. Long-term learning of novel words or digit sequences depends on STM, and performance in STM tasks is influenced by information in LTM. For example, although STM is predominantly phonological, performance in STM tasks is nevertheless influenced by lexical or semantic factors, or by other information stored in LTM. For example, words are recalled better than nonwords (Brener, 1940; Hulme, Maughan, & Brown, 1991), high-frequency words are recalled better than low-frequency words (Gregg, Freedman, & Smith, 1989; Hulme et al., 1997; Watkins & Watkins, 1977), concrete words are recalled better than abstract words (Bourassa & Besner, 1994; Walker & Hulme, 1999), sentences are recalled better than random word lists (Brener, 1940), sequences of letters are recalled better when they have a closer approximation to the statistics of English (Baddeley, 1964), and lists constructed from familiar sequences are recalled better than lists composed of unfamiliar sequences (Botvinick, 2005; Botvinick & Bylsma, 2005; Mathy & Feldman, 2012).

Some have interpreted this as evidence for a version of the activation view in which STM depends on activation in LTM language networks (Acheson, MacDonald, & Postle, 2011; Poirier, Saint-Aubin, Mair, Tehan, & Tolan, 2015). An alternative view is that although there is a separate STS, long-term semantic memory is activated in STM tasks, and the sustained semantic activation is fed back to STM to aid retention (Acheson, MacDonald, et al., 2011; Huttenlocher & Newcombe, 1976; Jefferies, Frankish, & Lambon Ralph, 2006a, 2006b; Jefferies, Frankish, & Noble, 2009; Patterson, Graham, & Hodges, 1994; Poirier et al., 2015; Savill, Metcalfe, Ellis, & Jefferies, 2015; Stuart & Hulme, 2000). This is sometimes referred to as semantic binding. Both of these possibilities run into exactly the same problems as the view that STM is activated LTM. Although almost all of the data comes from serial recall tasks, there is no indication of how simply activating semantic memory could code serial order.

The alternative view is that any benefit conferred by LTM occurs only at recall and operates by means of the kind of redintegration process described earlier (Schweickert, 1993). Redintegration is most commonly thought of as operating at the level of individual items. For example, a degraded mnemonic trace might be more likely to be reconstructed as a high-frequency word than as a low frequency word. Consistent with this view, factors such as lexicality, word frequency, and semantic information affect recall of item information but not order information (e.g., Poirier & Saint-Aubin, 1996; Saint-Aubin & Poirier, 1999b). However, Acheson, MacDonald, et al. (2011) reported a semantic effect on order recall. They combined a serial recall task with a secondary task requiring participants to make either semantic or orientation judgments about pictures. The semantic tasks led to more order errors than the orientation task, and produced more errors for word list than for nonword lists. They concluded that the semantic task interfered with the temporary activation of semantic representations within the language production architecture that could support STM. According to this view, nonwords would be unaffected by the nature of the secondary task as they have no semantic representations. Acheson et al. acknowledge that it may well be possible to explain their results in terms of redintegration, but they suggest that redintegration should operate at the item level and not benefit memory for order. However, the Bayesian framework presented by Botvinick (2005) was specifically designed to explain how information in LTM could help recover order information. In this framework, any information available from LTM, whether general LTM or a specifically linguistic memory, could potentially influence retrieval of both item and order information. Therefore it is still possible that their results could be explained by redintegration. From a psycholinguistic perspective, it would seem that listeners must necessarily form an integrated memory representation that combines phonological, syntactic and semantic information, although establishing exactly how those representations are stored and interact with each other remains a challenge.

A further form of interaction between LTM and STM happens at encoding. In the Baddeley and Hitch working memory framework, visually presented words must be recoded into a phonological form in order to be stored in the phonological store, and this recoding process must involve contact with representations in LTM to generate the necessary phonology. As least in the case of verbal STM, maintenance of information is helped by rehearsal which involves recycling information in STS. At each phase in the recycling process there is an opportunity for LTM to be involved in the reconstruction of degraded traces in STS. Furthermore, if items that are being rehearsed are thought of as being in the focus of attention, they must inevitably make contact with the same LTM representations that would be involved in the perception of those items.

Finally, long-term learning is not restricted to situations where there is an explicit intention to learn. Implicit learning occurs even in tasks which ostensibly only require STM. Long-term learning, and hence, access to LTM, must take place continuously if we are to be able to learn about the relationships between events occurring too far apart to be held within STM. For example, in the classic Hebb (Hebb, 1961) learning paradigm, performance in serial recall of lists of verbal material improves over successive repetitions. The implication of this is that LTM must be continuously engaged even in what might appear to be ‘pure’ STM tasks, so that some long-term learning can take place on the very first encounter with a list. If it did not, then every occurrence of the list would effectively be treated in exactly the same way as the first encounter and learning could never begin.

Taking these factors together, it is very difficult to imagine any set of circumstances in which LTM will not be involved to some degree in what might seem to be a pure STM task. As we will see below, the fact that LTM cannot simply be ‘turned off’ in STM experiments has implications for the interpretation of neuroimaging studies that have been presented as evidence against multistore models.

As STM and LTM have to perform similar functions, they are likely to share similar features. In particular, both involve basic processes such as encoding, retention, and recall. Both are subject loss of information through decay or interference, and both will be impaired when items to be remembered are similar. Brown, Neath, and Chater’s (2007) SIMPLE (scale invariant memory, perception and learning) model emphasizes these shared properties. Their model illustrates how a general principle of scale-independent similarity can be applied to simulate a range of key empirical findings in both STM and LTM without the need to make any distinction between STS and LTS. However, by focusing on the commonalities their model is unable to account for critical data such as the neuropsychological dissociations seen in STM patients or the change in the representational basis between STM and LTM. The success of the model is attributable to the fact that it embodies broad principles that apply to memory and perception quite generally. If these principles are indeed general they would be expected to apply equally well to separate short- and long-term stores. There is no contradiction between general principles and separate stores.

#### Cognitive models of STM as activated LTM

Atkinson and Shiffrin’s, 1971 paper is considered to be the classic statement of a two-store model. Figure 1A, based on the first figure in their paper, represents STS and LTS as separate boxes. Despite this they also say that “One might consider the short-term store simply as being temporary activation of some portion of the long-term store” (p. 83). More directly, Shiffrin (1975) wrote that “STS is the activated subset of LTS” (p. 214). Similarly, Norman (1968) talks about primary and secondary storage but also states that they “are different aspects of one large storage system” (p. 535). However, the theoretical force of these statements is unclear. Nothing seems to rest on this assumption and there is no discussion of its motivation or theoretical implications. This idea was developed further by Cowan (1988) who is regularly cited as supporting the idea that STM is activated LTM. Cowan stated quite clearly that “short-term storage should be viewed as an activated subset of long-term memory” (Cowan, 1988, p. 185), where short-term storage includes both the phonological store, and the visuospatial sketch pad. Additionally, in his model, a subset of activated LTM corresponds to the focus of attention (Figure 1C). However, on closer reading, much of what Cowan says has close parallels with the Baddeley and Hitch multistore model.[Fig-anchor fig1]

In later writings, Cowan and Chen (2008) state that “We thus propose that although the mechanisms of short-term memory are separate from those of long-term memory, they are closely related” (p. 104). They also acknowledge that “A phonologically based storage and rehearsal mechanism, such as the phonological loop mechanism of Baddeley (1986), may come into play primarily when items have to be recalled in the correct serial order” (p. 94) and “The phonological storage mechanism may be another instance of the use of the focus of attention to assemble a new structure in long-term memory” (p. 95). Cowan and Chen also appreciate that some extra mechanism is required to store novel information, such as serial order, that may not already be represented in LTM. In common with the WM model, Cowan’s model has a central executive which, among other things, is involved in “the maintenance of information in short-term memory through various types of rehearsal” (Cowan, 1988, p. 171). Cowan (2008) wrote that “Baddeley’s (2000) episodic buffer is possibly the same as the information saved in Cowan’s focus of attention, or at least is a closely similar concept.” At the very least then, Cowan’s model has the following components: LTM, an episodic buffer, a phonological store, and a rehearsal process controlled by a central executive. These would seem to correspond very closely to the components of Baddeley and Hitch’s (1974) multistore WM model. Given that not all short-term storage in Cowan’s model is supported solely by activated LTM, the crucial question then is what is the remaining force of the claim that STM is activated LTM? Is there any part of the process of retaining information over the short-term that can be served simply by activating LTM? One factor that makes it hard to answer this question is the absence of a computational specification of what it means for LTM to be activated, and of how that activation then supports memory. As Cowan’s position has evolved to accommodate a broader range of behavioral data, it has had to respect the fact that very little of that data can be explained purely in terms of activation. His theoretical position no longer equates STM directly with activated LTM.

Oberauer (2002, 2009) has proposed a three-state model which also gives a central role to activated LTM (Figure 1D). He makes an additional distinction between activated LTM and a direct access region. A similar three-state model has been proposed by Nee and Jonides (2013). Oberauer views LTM as an associative network in which related items can automatically activate each other. A subset of those activated items corresponds to a region of *direct access,* and items in the direct access region can be accessed immediately. Within the direct access region a smaller set of items is in the focus of attention. It is not clear whether the direct access region should be assumed to be a region of LTM. I will return to this question later.

#### Is there neuroimaging evidence for STM as activated LTM?

Historically, much of the debate as to whether there are separate long- and short-term stores has focused on whether various manipulations have different effects on long-term and short-term retention. For example, are there differences in the form of the serial position curve observed over different retention intervals? Wickelgren (1973) reviewed the behavioral data available in the early 1970s and argued that although the evidence favored the two-store account “the vast majority of all the cited evidence is worthless for making the distinction between short and long traces” (p. 426). Very little has changed since then. But might neuroimaging data be able to provide insights beyond those that can be had from behavioral data? If it could be shown that brain regions known to be responsible for LTM are activated during STM tasks, surely this would be evidence that those LTM regions were supporting STM? This unsound line of reasoning provides the rationale for the design and interpretation of a number of neuroimaging studies which have been presented as providing evidence that STM is simply activated LTM.

A flavor of the claims made in some neuroimaging papers can be found in the following quotations: “This result implies that activated long–term memory provides a representational basis for semantic verbal short-term memory, and hence supports theories that postulate that short- and long-term stores are not separate” (Cameron, Haarmann, Grafman, & Ruchkin, 2005, p. 643, abstract). “Temporary activation of long-term memory supports working memory” (Lewis-Peacock and Postle, 2008, title). “these findings support single-store accounts that assume there are similar operating principles across WM and LTM representations, and the focus of attention is limited.” (Öztekin, Davachi, & McElree, 2010, abstract). “Thus, the theoretical claim of our model—that maintenance in VWM entails the activation of long-term, speech-based representations—invalidates a central tenet of the multi-component model,” (Acheson, Hamidi, Binder, & Postle, 2011, p. 1359). Note that the first three quotations come from either the abstracts or the title of the papers so are presumably central to the claims being made by the authors.

Exactly what is the evidence that supports these forceful claims? Öztekin et al. measured hippocampal activity during a 12-item probe-recognition task and found that “activation of the medial temporal lobe was observed for all serial positions other than the last position, and that the activation level could be predicted from the underlying memory strength.” (Abstract). More generally, hippocampal activation decreased over item positions and this correlated with recognition accuracy. On this basis they claim that their “results are inconsistent with the traditional view that posits that there are distinct stores for WM and LTM” (p. 1130). This conclusion is based on what (Baddeley, 2003) has called the correlational fallacy; the assumption “that any variable that is broadly correlated with performance on the paradigm is crucial to it” (p. 729). As has already been shown, hippocampal activity is to be expected in STM tasks simply because LTM cannot be turned off in cases where it might not be needed. In fact LTM is very likely to be needed in this study, as a 12-item list will be well beyond the normal span of STM. Öztekin et al. favor an explanation based on Oberauer’s (2002, 2009) three-state memory model which posits that memory items can be in three states: a single item in the focus of attention, three or four items that are in an activated state in LTM but need to be retrieved from working memory, and items in a passive state which need to be retrieved from LTM. The first and last of these states are common to multicomponent models; one or two items may be in the focus of attention and some, especially in a 12-item list, will need to be retrieved from LTM (or possibly the episodic buffer component proposed by Baddeley, 2000). The critical question is whether these data provide any evidence that some items are in an activated state in LTM. As hippocampal activation is expected as a matter of course, even under the assumption that there is a separate STS, these data do not provide an answer.

Cameron et al. (2005) based their claim that activated LTM provides the representational basis for STM on data from an ERP study. They compared two conditions, one of which was assumed to involve memory for semantic information and the other was intended as a control task. In the memory task, participants saw a sequence of three words presented at a rate of one every 1000 ms, followed after 4000 ms delay period by a probe word. The participants’ task was to indicate whether the probe word was related to one of the three preceding words. In the control task, participants were required to indicate whether one of the words was unrelated to the other two, so in this case there was no memory load during the delay period. In both cases an incidental probe word was presented auditorily 2000 ms after the beginning of the delay period and this probe could be either semantically related or unrelated to the third word. The ERP analysis found an effect of semantic relatedness that was greater for the memory task than the control task, and this led the authors to conclude that STM was supported by LTM. This is a further example of the correlational fallacy. What the data show is a priming effect which is larger in the semantic memory task than in the control task. There is no evidence that whatever causes the sematic priming also plays a causal role in supporting STM. In the memory task participants are likely to be recirculating the to-be-remembered items. As items come to the fore they will be expected to elicit similar perceptual and semantic processes to those elicited at encoding.

Lewis-Peacock and Postle (2008) used fMRI combined with multivariate pattern analysis to establish whether a classifier trained to discriminate between three categories of stimulus (people, objects, locations) in a judgment task could classify neural activity patterns during the delay phase of an STM task. The judgment task involved answering questions such as how often the participants encounter a particular object. The classifier was trained on the data from this judgment task. In a second phase of the experiment participants learned to associate pairs of the items. In the STM task itself, participants were presented with a target stimulus for 1 s and, 11 s later, they saw a probe stimulus. Their task was to judge whether the probe stimulus was the associate of the target. During the delay period the classifier trained on the LTM judgment task could decode both the category of the target and the category of the probe above chance. Lewis-Peacock and Postle argued that “successful decoding of working memory activity would provide conclusive evidence for the activated LTM model” (p. 8768).

This is a further example of the correlational fallacy. The fact that LTM activity can be decoded during short-term retention interval does not imply that those LTM representations are responsible for short-term retention. Furthermore, the classifier was trained only to distinguish between representations of different semantic categories. It is quite possible that memory was supported primarily by some form of phonological storage in an STS, but Lewis-Peacock et al. only looked for semantic activation. Perhaps acknowledging this, Lewis-Peacock and Postle concede that “This model does not necessarily rule out a parallel contribution to delay-task performance by specialized working memory systems” (p. 8673). Data from a study by Shivde and Thompson-Schill (2004) suggests that this might well be the case. They used a similar STM paradigm where the task was either to judge whether the probe word was a synonym of the target or shared a vowel sound with a nonsense word probe. They found that the semantic task elicited delay period activation in bilateral inferior frontal gyrus and left middle temporal gyrus, while the phonological task elicited activation in left superior parietal cortex. In other words, when the task was phonological, the activation was in a region more commonly associated with phonological STM. Shvide and Thompson-Shill used univariate analyses so it is impossible to say whether a classifier trained on a phonological contrast would have been successful in decoding phonological information in the retention interval of their phonological task, or even possibly their semantic task. Not all brain regions that are activated will contain representations detectable by a classifier, and classification can often be successful in regions that show no univariate activation (e.g., Harrison & Tong, 2009). However, these data clearly show that the region that is activated during retention depends on what is being retained. There is more to the story than the simple claim that STM is activated LTM. Finally, a task requiring retention of a single item is one of the few that that could conceivably be performed solely by activating that item in LTM. The task demands might be too simple to be able to guarantee that a dedicated STS would be fully engaged.

Acheson et al. (2011) claim that “WM reflects the temporary activation of LTM representations” (p. 1359). They argue that “the theoretical claim of our model—that maintenance in VWM entails the activation of long-term, speech-based representations—invalidates a central tenet of the multi- component model” (p. 1359). However, their study really focused on a different and less contentious issue, which is whether short-term storage of verbal material depends on the same processes that are responsible for perception or production. Even adherents of the multicomponent view have considered the possibility that the phonological store might be isomorphic with the memory system supporting speech production (Page, Madge, Cumming, & Norris, 2007; Page & Norris, 1998a). Page and Norris (1998a) presented a connectionist implementation of their Primacy model of immediate serial recall which had close parallels to the speech production models of Dell and O’Seaghdha (1992) and Levelt (1992). Acheson et al. examined immediate serial recall of five-item lists using rTMS over either the MTG or posterior STG during a 3-s retention interval. MTG and posterior STG were chosen as they are implicated as the sites of two stages of production planning (Indefrey & Levelt, 2004). Their rationale was that if rTMS over these production areas impaired recall then these areas must be involved in STM. Despite the strong claims made on the basis of this data, the results are rather weak. rTMS had no significant effect on item order errors, and an influence on omissions only in posterior STG. Let’s pretend that there was a large effect of rTMS on both item and order errors in both regions. What might we conclude? At the core of the Working Memory model is a speech-based articulatory rehearsal process. Unless participants are actively prevented from doing so, they will almost certainly be trying to rehearse the five-item list during the 3s retention interval. rTMS that interfered with production should also interfere with rehearsal. The only surprising finding here is that the effects were not larger than they actually were.

A positive feature of the Acheson et al. study is that they used a standard serial recall task. Serial recall is the most widely used task to study short-term verbal memory and is the primary target for computational models of verbal STM. The other neuroimaging studies described here used nonstandard tasks that do not have a well-established relation to specific components of memory in multistore models. For example, Lewis-Peacock and Postle used a retention interval of 11 s, which is well beyond the duration of the phonological store. Indeed, this seems to be a common problem. Talmi, Grady, Goshen-Gottstein, and Moscovitch (2005) tried to make a case against the multistore models using data from a study where participants had to remember lists of 12 words. This is twice the normal span for words. The choice of task may in part be determined by the difficulties associated with using standard laboratory tasks in a neuroimaging environment. However, it is technically possible to run standard immediate-serial recall experiments using fMRI (e.g., Kalm, Davis, & Norris, 2013).

Öztekin et al., Cameron et al., and Lewis-Peacock et al. have all made the mistake of assuming that the presence of neural activation of brain regions normally associated with LTM during an STM task implies that long-term representations in those regions form the causal basis of STM. They have all committed the correlational fallacy and failed to appreciate Atkinson and Shiffrin’s point that “both STS and LTS are active in both STS and LTS experiments.” The only way to construct a model of STM where there would be no LTM activation in a STM experiment would be by assuming that information in STS was stored in opaque containers such that the contents of that store were inaccessible to other processes. If information in STS is visible to other processes then it should activate those processes too.

#### What is activation?

If STM is supported exclusively by activated LTM, it seems reasonable to ask what computational function is performed by activation that would enable it to encode, maintain, and retrieve information from STM. This is a fundamental and largely unrecognized problem with all models invoking activated LTM. Although the core explanatory concept is activation, there is no explicit definition of what activation means. In the memory literature the term activation often refers to the deployment of a limited capacity resource that can be used to support WM (Anderson, 1983; Cantor & Engle, 1993; Just & Carpenter, 1992). However, there is no computational definition of activation of LTM that would explain how that ‘activation’ might be sufficient to maintain representations in STM. As Phillips (2003) noted in his commentary on Ruchkin et al.’s (2003) position paper, sometimes activation was used “in a psychological sense, sometimes to refer to EEG measures, and sometimes to refer to underlying neuronal activity” (p. 752). Worse still, in the absence of an explicit computational model there is no agreed psychological sense of *activation* to fall back on; it simply is not at all clear what it means to say that STM might be activated LTM. Without an understanding of the computational functions served by the neural activation seen in LTM systems, any attempt to interpret correlated activity in brain regions associated with LTM during short-term retention remains highly speculative. We know too little about how to interpret neural activation for it to form the basis of an explanation. Even if we knew which neural processes caused activation, this would not tell us what computational function was being performed by those processes.

A further problem for activation-based theories is that recent neuroimaging work suggests that storing information in STM may not depend on sustained neural activation of any form. Several studies (LaRocque & Lewis-Peacock, 2013; LaRocque, Riggall, Emrich, & Postle, 2016; Lewis-Peacock, Drysdale, Oberauer, & Postle, 2012; Sprague, Ester, & Serences, 2016) have tried to address the question of whether STM is supported by sustained neural activation. These studies all used a retrocuing paradigm combined with multivariate pattern classification. In an initial phase of the experiments a classifier was trained to discriminate between different stimuli that would have to be remembered. For example, in the LaRocque et al. (2016) study participants had to remember the direction of motion of arrays of dots. The pattern classifier was then used to track the progress of activated memory representations over the course of a retention interval while participants shifted their attention between one of two stimuli that might have to be remembered. In the critical conditions, two visual items are presented along with a cue indicating which item to attend to. There is then a delay period of 16s. Halfway through the delay period a retrocue is presented to indicate whether the participant should maintain attention on the same attended item or switch attention to the previously unattended item. A test probe is then presented at the end of the delay period. Performance on the test probe is high regardless of which item is probed. All of the studies reported that during the first half of the retention interval, only the item that was attended to could reliably be decoded by the pattern classifier. However, when the retrocue indicated that attention should be switched to the previously unattended item, then that item could now be decoded. That is, only attended items could be decoded but it did not matter whether those items had previously been in an unattended state where their representations could not be decoded. LaRocque, and colleagues (LaRocque & Lewis-Peacock, 2013; LaRocque et al., 2016; Lewis-Peacock et al., 2012) concluded that only items that were in the focus of attention were activated, and that sustained activation was not necessary to maintain items in STM. Sprague et al. describe the unattended information as being stored in latent or activity-silent codes. In other words, not only is there no evidence that STM involves sustained LTM activation, but it may not require sustained activation of any form at all over intervals of up to 16s. In reviewing some of this data LaRocque, Lewis-Peacock, and Postle (2014), two of whom had previously argued that STM was activated LTM (Lewis-Peacock & Postle, 2008), quite reasonably pointed out that “that this result should be taken as a call for clarification of what is meant by “activated.” . . . Rather than referring to such items as being retained in the “activated” portion of LTM, a word more in keeping with the function of STM—such as “accessible”—might be more appropriate to describe STM items outside the FoA” (Focus of Attention. p. 9). However, the shift from ‘activated’ to ‘accessible’ completely eliminates the theoretical force of any claim about the role of LTM in supporting STM. Without further clarification, the term ‘accessible’ does little more than redescribe the data.

Part of the problem with explanations based on activation may stem from the tempting allure of the activation metaphor. Activation has been a popular theoretical metaphor in psychology since the 1960s. In the context of memory models, activation usually refers to the strength of a representation, and activation levels are inferred from how well an item is remembered. With the rise of connectionist or neural network models in the 1980s, activation became even more central to psychological thinking. Activation is the currency of communication between the artificial neurons. Connectionist models had the great advantage of producing quantitative simulations that could be directly tested against the data. The concept of activation in these models has clear computational underpinnings. However, it sometimes seems that the successful use of activation in connectionist modeling has been taken to license indiscriminate use of the term in verbal models.

#### Computational models

In any scientific endeavor, progress depends on developing theories that make clear and testable predictions. In STM research much of that progress has been driven by computational modeling. For example, the basic principles of Baddeley and Hitch’s WM model have been formalized in computational models of the phonological store (Burgess & Hitch, 1992, 1999, 2006; Page & Norris, 1998b). These and other computational models of STM (e.g., Botvinick & Plaut, 2006; Brown et al., 2000; Farrell, 2012; Farrell & Lewandowsky, 2002; Henson, 1998) produce accurate quantitative simulations of most of the benchmark data from STM tasks: the form of the serial position curve, the nature of the errors in serial recall, effects of perceptual grouping, complex patterns of phonological similarity effects, sequence learning, and much more. One could argue that the problem with these models is that they are so good that it is becoming increasingly difficult to devise experiments to distinguish between them. Nevertheless, these are all concrete models that can be subject to empirical scrutiny. In contrast, there are no computational models based on activation of LTM. Before activation-based models can be taken seriously they need to be clearly formulated and to be shown to be competitive with existing computational models.

#### STM must be able to represent multiple tokens

The spoken sentence “Buffalo buffalo buffalo Buffalo buffalo” contains five tokens of the same phonological word form – ‘bΛfələʊ. If you do not know that a buffalo is a North American bison, that ‘to buffalo’ means ‘to confuse,’ and that there is a town called Buffalo, this sentence will probably leave you completely buffaloed.^1^ In fact, even if you know all three of those it’s still hard to understand. Nevertheless, if you hear this sentence it is very easy to repeat it back—it’s just the same word repeated five times. If STM were nothing more than the activated portion of LTM then a sentence or list containing five repetitions of the same phonological word form could not be recalled. There would just be a single very strongly activated representation of bΛfələʊ.

This is an example of what Jackendoff (2002) called ‘the problem of two’ – the cognitive system needs to be able to represent multiple tokens of the same type.^2^ Of course, it might be the case that LTM contains multiple representations of the word ‘buffalo’ that can each be activated separately. But how many? Is it possible to repeat the sentence “Buffalo buffalo Buffalo buffalo buffalo buffalo Buffalo buffalo”?^3^ Maybe LTM contains a whole herd of buffalo? Possibly these sentences could be coded by a single LTM representation with either five or eight units of activation. But what about a list like “buffalo cowboy cowboy buffalo”? Could activation levels reliably differentiate between the lists “buffalo cowboy cowboy buffalo” and “cowboy buffalo buffalo cowboy” so that they could be recalled in the correct order? The idea that STM is nothing more than activated LTM fails to provide a solution to ‘the problem of two.’ Even the simple requirement to remember sentences or lists containing two occurrences of the same type (word) cannot be supported solely by activating LTM. In the memory literature the importance of being able to represent multiple tokens was highlighted by Potter (1993) in discussing what she called “Very short-term conceptual memory.” She pointed out that ”It is not enough just to activate the type representation of each word in LTM; one must set up an episodic representation that includes a token of each word type, tokens that are pointers to or copies of the long-term representation of the words and their meanings” (p. 159). Note that the need to represent multiple tokens is not limited to verbal processing. In the context of visual perception and attention Kahneman and Treisman (1984) made a distinction between “episodic tokens and semantic types” (p. 55) in the development of their concept of an object file. Theoretical accounts of phenomena such repetition blindness (Kanwisher, 1987, 1991) and the attentional blink (Bowman & Wyble, 2007) also require a distinction to be made between type and token representations.

Most computational models of STM have the ability to represent multiple tokens. One straightforward way of achieving this can be found in connectionist models of immediate serial recall that are based on forming associations between item representations and representations of list position (e.g., Burgess & Hitch, 1992; Brown, Preece, & Hulme, 2000). The Burgess and Hitch model was explicitly developed as a model of the articulatory loop component of Baddeley and Hitch’s (1974) working memory model. In their model a vector representing list position slowly evolves so as to change slightly as each successive word is presented. At each position, the network learns an association between the position vector and the word. At recall, the states of the position vector can be replayed and successive states of the vector will tend to reactivate the word in the list associated with that position. Because the same word can be associated with more than one position, the model has no difficulty in remembering sequences where words are repeated. One might suggest that the words that are activated must be in LTM and this could then be construed as performing short-term storage by activating LTM. However, the crucial point is that the model’s performance depends entirely on the position-item association mechanism which exists purely to perform short-term serially ordered recall. STM is supported by a dedicated mechanism which exists in addition to LTM. That mechanism is a STM store. Furthermore, in this particular model, the memory is encoded in the weights between the position vector and the word representations and not by sustained activation over LTM. This reminds us once again that although there is a close relationship between LTM and STM, LTM cannot do the job of supporting STM on its own. The problem of two poses an insurmountable barrier to the idea that STM might be supported simply by activating LTM. This holds regardless of the content of STS. Exactly the same argument will apply to STM for semantic information (Martin & Romani, 1994; Martin, Shelton, & Yaffee, 1994; Shivde & Anderson, 2011) or visual information.

#### STM must contain structured representations

The ability to represent multiple tokens is a basic prerequisite for a simple passive STM system. However, this is not sufficient for a memory store that could serve as a mental workspace for WM. A consequence of being able to actively manipulate the contents of WM is that this can create completely new structures which have never been encountered before, which can themselves be manipulated. By definition, this productivity cannot be captured simply by activating preexisting representations (Logie, 2003; Logie & Della Sala, 2003). In simple short-term verbal memory tasks the only novelty is generally in the ordering or composition of the items to be remembered. In contrast, WM has to have the capacity to store a vast and unpredictable set of representations. There might be some STM tasks that can be performed simply by activating preexisting structures, but rarely will that be possible in a WM task.

#### Variable binding

To hold structured representations of objects or scenes the system must also be able to perform variable binding (see van der Velde & de Kamps, 2006, for review). For example, consider how we might maintain a coherent representation of a sentence such as “The young boy saw the boy who was singing.” Here the problem is not simply representing the order of the words, or even that there are two tokens of the word boy, but appreciating that there are two different boys, one of whom is singing and one of whom is young. It’s necessary to both represent multiple tokens and the bindings between each of those tokens and other components of the sentence. However, this cannot be achieved solely by coactivating and associating existing representations (as assumed by Cowan & Chen, 2008). This can be appreciated by considering the simple case of forming associations between AB, BC, and CA. Associating these three pairs will necessarily form associations between all items in the triple ABC. It will therefore be impossible to determine whether what has been learned was AB, BC, and CA, or ABC. What is needed are additional representation of the individual conjunctions (e.g., creating an ‘AB’ node in a connectionist network). In STM this could be achieved by variable binding. That is, STM would contain a set of variables to which arbitrary representations could be assigned. For example, *x* = (A, B), *y* = (B, C) would construct bound representations assigned to the variables *x* and *y*. This process may or may not activate representations in LTM, but, for the reasons already given, it should not form direct associations between A and B or between B and C. This might seem to present a severe problem for Oberauer’s three state model shown in Figure 1D.

Oberauer depicts his model as having three concentric regions—activated LTM, the direct access region, and the focus of attention, all of which select a different subset of LTM representations (shown by the lines and nodes). However, while the diagram and the term *region* imply that the direct access region is simply a subset of activated LTM, the operations taking place within the direct access region involve much more than activation. According to Oberauer “the region of direct access is a mechanism for establishing and holding temporary bindings between contents. . . . By supporting arbitrary bindings between virtually any content with any context, this system enables the compositionality of thought that many theorists regard as a hallmark of human cognition (Fodor & Pylyshyn, 1988): we can create an unlimited number of different ideas by freely combining content elements into new structures.” (Oberauer, 2009, p. 53). The direct access region is therefore much more than just an activated *region* of LTM. It must have all of the computational machinery needed to support all aspects of higher cognition, and to have the same functionality as the central executive and episodic buffer components of the Baddeley and Hitch WM model. As with Cowan’s model, it is not clear whether activated LTM alone plays an essential part in the model’s operation, or in its ability to retain information over the short-term. The fact that Oberauer’s model needs to be supplemented with a binding mechanism reinforces the argument that a model of STM needs more than just activated LTM to make it work. Indeed, the big challenge for any model that might be based purely on activation of LTM would be to make it work.

#### Visual STM

The case for STM as activated LTM has been advanced predominantly in the domain of verbal STM. Perhaps this is because some verbal STM tasks might be seen as simply requiring the participant to remember a collection of familiar words, which might be achieved simply by activating existing representations. As we have seen though, the need to remember words in sequence demands that words be bound to positions. However, it has long been appreciated that perception of visual objects depends on forming structured representations that bind together features such as shape, color and location (Kahneman & Treisman, 1984; Marr, 1982; Pylyshyn, 1989, 2001). Not surprisingly, the notion that STM might simply be activated LTM has been less influential here. Combinations of visual features can produce completely novel visual objects that cannot possibly have preexisting representation in LTM. Nevertheless, configurations of complex objects can be stored in STM. Even an image as simple as an array of randomly placed identical dots cannot be represented just by activating the appropriate number of dots. The dots must each be individuated and bound to a unique spatial location. Indeed, the change detection task used in many studies of visual STM (e.g., Luck & Vogel, 1997) requires participants to judge whether an object (such as a colored square) in a particular location has changed color. There may well be other objects of the same color in other locations. The task can only be performed by binding colors to locations. The task of remembering a spatial sequence presents even more of a challenge for activation accounts. The Corsi block task (Corsi, 1973) requires participants to touch a series of blocks in the same order as the experimenter had done. Here there is no need to remember the spatial location of the blocks, as they remain in view; instead, the participant must remember the binding between the location of the blocks and their order.

In the neuroimaging literature on visual STM many of the data are consistent with what the ‘sensory recruitment’ hypothesis (Lee & Baker, 2016; Serences, Ester, Vogel, & Awh, 2009). This suggests that maintenance of information in visual STM is achieved by recruitment of the same neural mechanisms that are engaged in encoding that information. That is, information in STM is stored in perceptual areas and not in those areas involved in LTM. The sensory recruitment hypothesis is supported by data showing that information maintained in visual STM can be decoded from brain regions known to be involved in visual perception (Harrison & Tong, 2009; Serences et al., 2009). Information maintained in visual STM can also be decoded from regions of parietal cortex (Bettencourt & Xu, 2016; Christophel, Hebart, & Haynes, 2012; Sprague, Ester, & Serences, 2014; Sprague et al., 2016) similar to those that that have been found to be active in verbal STM. However, at the moment it is far from clear how the neuroimaging data can be mapped onto cognitive models of visual STM. For example, some have argued that there is more than one visual STM. Basing his argument primarily on neuropsychological data Logie (2003), has suggested that there may be one store for spatial layout, and one that supports the ability to manipulate mental images, and to retain dynamic information such as sequences.

#### Copies or pointers?

In Atkinson and Shiffrin’s modal model the store is a box that you put things in. But what do you put in the box? This was never explicitly specified. In their 1966 paper they suggested that the item “that is placed in the buffer may be considered to be an amount of information which is sufficient to allow emission of the correct response” (p. 3). One way to achieve this is to store a copy of a representation of the input. For example, in the WM model the primary responsibility for storage of verbal information falls on the articulatory loop, which stores copies of phonological representations. Alternatively, instead of storing copies, one might store pointers to representations stored elsewhere. Either would be sufficient to allow emission of the correct response.

The possibility that an STS might be based on pointers rather than copies has a number of interesting implications. If the pointers address representations in LTS then this would greatly complicate the relationship between STS and LTS. However, pointers might also operate within the bounds of a self-contained modality-specific STS. As we will see, the advantage of this option is that it can make very efficient use of the available storage capacity. Far from blurring the lines between STS and LTS, consideration of how a pointer system might work actually reinforces the case for modality-specific short-term stores.

Perhaps the simplest possible model of an STS is Conrad’s (1965) slot model—STS would contain a number of slots, and each slot would contain a copy of an item to be remembered. But what does it mean to store a copy? Exactly what is it that’s copied into memory? In principle, when trying to remember ‘dog,’ we could store a copy of everything we know about the word ‘dog.’ That might seem excessive. More plausibly, we might store only a subset of the information held in LTM; perhaps a phonological representation of ‘dog.’

An alternative to storing copies of objects is to store pointers to representations elsewhere. As every programmer knows, it is usually much more efficient to manipulate pointers than to copy entire data structures. In computing terms a pointer is the address of a location in memory where information is stored. The great advantage of using pointers is that the pointers generally take up much less storage than the data they point to, and they eliminate the need to copy the data. For example, if the data objects were lexical representations of words, any list of words could be represented economically by a list of pointers to those objects. The STS storage requirements would remain the same no matter how complicated the lexical representations were. Importantly, STS could represent multiple tokens by having more than one pointer index the same type representation (the same word in LTS). Thus, if the capacity of STS happened to be five pointers, it would be possible to retain the identity and order of any five objects.

It is worth bearing in mind that it is possible to write two programs to perform the same computations that differ solely in terms of whether they rely on copying data or manipulating pointers. Indeed, programming languages differ in terms of whether they perform assignment by copying data or pointers. In C, “*a* = *b*” copies the data in *b* to *a*. In Python, “*a* = *b*” sets *a* to point to the same data as *b*. If one were to write two superficially identical programs, one in C and one in Python, they would produce the same output, but the underlying operation of the programs would be different.

Some of the confusion seen in the neuroimaging literature might come from an assumption that a distinct STS would hold representations copied from LTM, and that therefore LTM should not be activated while that information is being retained in STM. Interestingly, in computational models of STM, what is stored is generally more akin to a pointer than a copy. In the Burgess and Hitch model the weights between position vectors and word vectors act as pointers to a single representation of a word. There is no need to have multiple copies of a word.

#### Pointers, address space, and efficiency of storage

Although it is tempting to think that memory capacity might be determined by the number of available pointers, not all pointers are created equal. The amount of memory required to store a pointer will vary as a function of the number of objects that the pointer can potentially address. In an early 8-bit computer an 8-bit pointer could only address one of 256 memory locations. A 16-bit pointer could address one of 65536 locations. How large a pointer might be necessary in order to address any possible representation that might be stored in LTM? Let’s pretend that a 32-bit pointer would be sufficient as that could address any one of 4.3 * 10^9^ representations (on the assumption that each representation corresponds to a single address). But an 8-bit pointer would be more than sufficient to index the set of phonemes in a language. With a memory capacity of 32 bits it would therefore be possible to store pointers to 4 phonemes, or just a single pointer to any object in LTM—which might be a phoneme. There’s a simple trade-off. For a given memory capacity it is possible to store pointers to either a large number of items selected from a small set, or a smaller number of items selected from a larger set. This has implications for how best to make efficient use of available memory capacity. Imagine that there is some set of computations in language comprehension that only requires manipulation of phonemes. If those computations can be performed in a dedicated speech store that only needs to reference speech representations, then the total memory requirement (in bits) will be much less than if those same computations have to be performed in a general purpose system that has the potential to reference all possible representations in LTM. Note that a specialized store could be made even more efficient if it could take into account the redundancies of the language to construct a compressed representation. One estimate is that each phoneme in English phoneme could be coded by as few as 3 bits (Stilp, 2011). That is, a dedicated store might be able to store as much as 10 times as many phonemes in a given capacity than a general-purpose store. Note that the amount of data compression possible will depend how much context can be taken into account (Shannon, 1951). Nevertheless, a dedicated store that incorporated knowledge of say, syllables,^4^ or the phoneme transition probabilities of English, could make far more effective use of storage than one that had to rely on pointers to LTM. One point in favor of a phonological store like that in the WM model is therefore that it would make very efficient use of available memory capacity.

Note, however, that if we used an 8-bit pointer to represent phonemes in a phonological store, that pointer could not make direct contact with phonological representations in LTM on its own. If LTM had, say, a 32-bit address space, an 8-bit pointer could only address a subset of that space. A simple solution would be that a modality-specific store could add a bit pattern to the modality-specific pointer that would serve to select the relevant subset of LTM representations. In our simple example, the phonological store would pad out the 8-bit pointers to 32-bits with an extra 24 bits that effectively say “I’m a phonological representation” and ensure that the 8-bit pointer can address the correct part of LTM. In the brain, if phonological representations in LTM were, in a sense, stored close together, then connections from phonological STM could target specifically those representations. The same reasoning holds even if what is stored are copies. Efficiency will be greatest if only the minimum amount of information required is copied into the store. Although the pointer argument is made from a purely computational perspective, an analogous argument can be made from the perspective of neural economy (Bullmore & Sporns, 2012). If some computations involve a restricted set of processes and representations, then the neurons responsible for those computations should be strongly interconnected and physically adjacent so as to minimize expensive long-range connections.

All of these arguments will apply equally well to any system that implements the same functionality as pointers. For example, in a connectionist model relying on weighted connections between nodes, the greater the number of representations that can be stored, the more nodes and connections will be required.

#### Pointers and single-store models

Proponents of single store models have also invoked the idea of pointers. In arguing that working memory is a state of activated long-term memory Ruchkin et al. (2003) say that “Prefrontal cortex provides the attentional pointer system for maintaining activation in the appropriate posterior processing systems.” (abstract). That is, there is an attentional pointer system that takes care of all of the processing operations that are needed by an STS, whereas the representations that are being retained exist only in activated LTM. Ruchkin et al. do not specify exactly what is meant by an attentional pointer system, nor by activation. However, adding a pointer system to activated LTM at least has the potential to overcome the fundamental barrier that undermines simple activation models - the problem of two. But exactly how does such a model differ from one that posits separate short- and long-term stores? The pointer system functions as a store and must necessarily be responsible for representing multiple tokens and serial order. The claim seems to be predominantly one about neural implementation or localization rather than function; the pointers are in prefrontal cortex and the representations are in posterior systems. As I have already argued, pointers will generally provide the most efficient way to represent multiple tokens and to manipulate information in STM. However, there are also efficiency gains to be had by restricting pointers to operate on representations held within a local STS rather than using a single pointer system that can address all of LTM. However, apart from the efficiency argument, a greater concern is that, under this view, the basic behavioral phenomena that motivated the WM model remain unexplained. A pointer-based store that could address any representation in LTM would have no reason to be modality-specific - the pointers could equally well address phonological, semantic, or even visual representations. So why does verbal STM seem to be primarily based on a phonological code, and why does phonological coding of visual input appear to be eliminated by articulatory suppression? Why would the pointers point to purely phonological representations if they could equally well point to lexical representations?

Ruchkin et al. say that “findings of multiple, modality-specialized short-term stores are fully compatible with the position that short-term memory corresponds to activated long-term memory representations, given that long-term memory involves multiple, modality-specialized stores.” (p. 712). However, the behavioral data show that the qualitative behavior of the different stores is different. At the very least they have different time-courses. If short-term retention is controlled by a single pointer system, then all short-term storage should have the same time-course.

Most of the data that Ruchkin et al. martial in support of their view come from electrophysiology. However, in common with the neuroimaging data reviewed above, this is entirely correlational and shows only that, during STM tasks, there is neural activity in brain regions usually associated with LTM or perceptual processes. We have already seen that, according to a multistore view, LTM and perceptual regions should also be activated, so finding correlated activation does not tell us whether that activation plays a causal role in retention. The only data we have that speaks directly to that question is the data from STM patients.

Ruchkin et al.’s position makes it clear that we should not view the question of whether there are distinct long-term and short-term stores as being a simple dichotomy. There is a very large space of possible models. The simplest possible model might seem to be Atkinson and Shiffrin’s modal model, but even there the STS could contain copies, or could contain pointers. In most cases these two alternatives would produce indistinguishable behavior. Pointers might address representations in LTM, or representation in modality-specific stores. A short-term phonological store might operate with pointers to representations held within that store itself. Additionally, it might be a specifically mnemonic store, or parasitic on processes responsible for perception or speech production.

An additional layer of complexity arises when we consider how any of these memory systems might be implemented in the brain. For example, if there is an STS containing copies of information in LTM, will the binding between the two sets of representations elicit neural activity? Or, to put it the other way around, what does neural activity tell us about the computations involved? More fundamentally, what is the mechanism of neural binding underlying STM? The space of possibilities grows even larger when we move from just considering STM to thinking about working memory and how to manipulate representations and coordinate them across different modalities. Note that I have referred to a system holding pointers to LTM as an STS even though the representations are in LTM. If there is a system where the STS contains pointers to LTM, should we really call this a STS, or is it just a pointer system? My own inclination is to stick with the term STS, as the pointers are doing all of the hard work. They also control the capacity of STM—more pointers means greater capacity.

#### Summary

When reading many of the papers cited here it is easy to form the impression that there is a clearly formulated model of memory in which STM is simply the activated portion of LTM. No such theory exists. This should not really come as a surprise as such a model would founder on the problem of two. Any model of STM must have some additional mechanisms beyond simple activation. There must be mechanisms to control the activation, to store multiple tokens, to recall those tokens in the correct order, and to create novel structured representations that cannot yet be in LTM. Only then could such a model begin to compete with existing computational models of STM developed within a multistore framework. However, those additional mechanisms would effectively amount to a dedicated STS. In a system relying on storing pointers in a STS, STM would indeed depend on LTM representations, but all of the heavy lifting would be done by processes outside of LTM itself. The theories of Cowan and Oberauer, which have often been cited as models where STM depends on activated LTM, have additional mechanisms to support binding and serial recall. Indeed, serial recall should probably be seen as the natural task to investigate the relation between verbal STM and LTM. A simpler task where there is no need to represent order might conceivably be performed largely using LTM. Recalling the words “buffalo cowboy cowboy buffalo” in the correct order requires a memory for multiple tokens, but judging that the list contains the word “cowboy” does not. It is also important to bear in mind that factors such as phonological similarity can have different effects on memory for order information and item information. Although phonological similarity generally has a detrimental effect on recall of order, it can have a beneficial effect on item memory (Fallon et al., 1999; Gathercole, Gardiner, & Gregg, 1982; Gupta, Lipinski, & Aktunc, 2005).

Although we can be sure that an activation-based single store model will not work, representations in LTM have an important role to play in assisting STM. One of the big questions here is whether that assistance takes the form of a continuous interaction between LTM and STM during retention, or a redintegration process operating only at recall. Note that if activation from LTM is assumed to improve retention of order, that source of activation needs its own ordering and binding mechanism. An even bigger question though is “What is stored in STM?”. The primary options are copies and pointers. But copies of what, and pointers to what? It is clear that what is stored in the phonological store of the Working Memory model are copies of speech-based representations. However, in the Burgess and Hitch model which is intended as an implementation of the phonological store, the weights can be thought of as pointers.

Interestingly, the computational properties of this model would remain unchanged if it were assumed that the item representations that were pointed to were in LTM rather than a separate STS. That is, the change would simply be in terms of how the models were described or interpreted, or how they might be implemented in the brain, not in terms of how they worked. As noted earlier though, there are advantages to using pointers within a dedicated STS. Using pointers to local representations can make economical use of storage. It could also minimize the need for information to be transferred between different brain regions. This would be advantageous given that most of the connections within the brain are local. Together, these engineering constraints make a case for expecting the existence of modality specific stores. That is, while it may seem more parsimonious to have a single store, it may be more complicated to build one.

In experimental psychology, much of the progress in understanding STM has followed from the development of computational models. Before the advent of computational theories the Working Memory model had no proper account of how serial recall was actually performed, even though much of the data that motivated the model came from the serial recall task. However, models such as those of Burgess and Hitch (1992), and Page and Norris (1998b) are implementations of the phonological store component of the WM model. Those models can simulate a wide range of data form serial recall. Theories making claims that STM is supported by activated LTM need to rise to the same challenge. This is not simply a matter of requiring them to simulate the data, the models need to be specified in more detail to even begin understand what claims about activated LTM actually mean.

I have concentrated primarily on one possible class of single-store model, those relying solely on activation. Might it be possible to produce more complex single-store models that wouldn’t fall foul of the problem of two? The pointer-based models of the form proposed by Ruchkin et al. could represent multiple tokens, but only by adding extra mechanism that serves all of the functions of an STS. However, such a model still faces the seemingly insurmountable challenge of accounting for the neuropsychological data from STM patients. Ruchkin et al. tried to downplay the significance of the neuropsychological data by suggesting that patients with deficits in phonological STM deficits “should be indicative of impairments in establishing long-term memories for those representations” (p. 711). In other words, this is an attempt to turn the argument presented earlier on its head. The problem phonological STM patients have in long-term learning of novel words is not because they have poor STM but because they have trouble establishing phonological representations in LTM. Given that the claim is that STM is supported by activated LTM, it is not clear why a problem in establishing new representations should prevent those representations from being activated—which should be all that’s necessary to hold information in STM. According to the Ruchkin et al. view, language comprehension should also depend on activation of phonological representations in STM. However, STM patients have good comprehension abilities, suggesting that they have no problems in activating those representations. Furthermore, the patients’ primary STM deficit is in remembering sequences of familiar items such as words or digits. This should not place any demands on establishing new representations in LTM. Additionally, these patients definitely can establish new representations of verbal material in LTM because they have little trouble with paired-associate learning.

More recently, Surprenant and Neath (2009) also presented a case for a single store model. They mounted a spirited attack on nine lines of argument that have been used to support the distinction between STM and LTM. Number nine concerned data from patients with intact STM but impaired LTM, but they offered no explanation of how data from STM patients might be accounted for in a single store model.^5^

## Conclusions

The primary argument advanced here is that any STM system must be able to store complex representational structures that have never been encountered before, whether this be novel sequences of words, or novel combinations of visual features. By definition, such representations cannot already exist in LTM, and therefore cannot be stored simply by activating LTM. The most basic requirement for any memory system is to be able to store multiple tokens of the same type. This is essential for even a simple STM task such as serial recall. If LTM is a store of type representations, STM cannot function simply by activating LTM types. Of course, it is possible to argue that LTM has many representations of a given type, but this would be grossly inefficient as it would imply that all representations in LTM are extensively duplicated. The standard solution is to allow memory to either contain multiple copies of representations, which ensures that only those representations being retained at the moment are duplicated, or to contain pointers to the representations of single types in LTM; the same representation in LTM can be pointed to by multiple pointers in STM. However, simple pointers from STM to LTM are not sufficient to solve the binding problem. There must also be provision for the construction and storage of more complex representations, and this must involve something equivalent to variable binding. Whatever is going on when we store information in STM is far more elaborate than activating LTM, or putting copies in boxes. The important question is not simply whether there are separate short-term and long-term stores, but what is stored in STM. If STM contains pointers, the information capacity of STM will depend on the address space—the wider the range of LTM representations that can be stored in STM (pointed to) the more bits will be required to hold those pointers. This provides an additional motivation for modality-specific stores of the form incorporated into Baddeley and Hitch’s working memory model. If some set of computations only need access to a limited range of information that has to be stored temporarily (say phonological information) then it will be more memory-efficient to process that information in a store that is specialized to hold only that kind of information.

The suggestion that STM might be nothing more than activated LTM may have the merit of parsimony, but it simply doesn’t work. A simple activation process would be unable to solve the problem of two, or to store novel representations. It follows that any model that places an emphasis on storage by activated LTM must be supplemented by some additional mechanism that can represent multiple tokens serial order. That mechanism must be able to perform the variable-binding operation required to construct novel representations. That additional mechanism would then amount to what has conventionally been thought of as a short-term store. In fact, the resulting model would look very much like existing computational models of STM. Some may still prefer to describe this by saying that STM is activated LTM. If they make it clear that there must be some additional mechanism, and explain how that mechanism operates, at least we’ll know what they mean.

## Footnotes

1 The sentence should be interpreted as indicating that bison who are from the town of Buffalo confuse (buffalo) other bison from the town of Buffalo. It is commonly used as an example of several different linguistic phenomena and may have been generated independently by a number of researchers. See http://www.cse.buffalo.edu/~rapaport/buffalobuffalo.html

2 The same argument applies even if ‘Buffalo’ and ‘buffalo’ are assumed to activate separate semantic representations and therefore correspond to different types as there will still be multiple tokens of the those types.

3 See https://en.wikipedia.org/wiki/Buffalo_buffalo_Buffalo_buffalo_buffalo_buffalo_Buffalo_buffalo for interpretation.

4 Simply coding the input in terms of syllables rather than phonemes (allowing for the fact that there are many more syllables than phonemes) can reduce the requirements to between 3 and 4 bits per phoneme.

5 See Thorn and Page (2009) for a more detailed rebuttal of the Suprenant and Neath arguments.
